# Objective Sleep Measures and Cognition in Middle-Aged and Older Adults: A Cross-Sectional and Longitudinal Analysis in the ALBION Study

**DOI:** 10.3390/medsci14030340

**Published:** 2026-06-23

**Authors:** Angeliki Tsapanou, Artemis Margoni, Eirini Pavlou, Eva Ntanasi, Eirini Mamalaki, Elias Manolakos, Mary Yannakoulia, Nikolaos Scarmeas, Christopher Papandreou

**Affiliations:** 1 Aiginition Hospital, Department of Neurology, National and Kapodistrian University of Athens, 11528 Athens, Greece; e.ntanasi@hotmail.com (E.N.); eir.mamalaki@gmail.com (E.M.);; 2 Master’s Programme in Biostatistics and Health Data Science, Department of Mathematics, School of Medicine, National and Kapodistrian University of Athens, 15784 Athens, Greece; artemar@med.uoa.gr; 3 Department of Neuropsychology and Cognitive Rehabilitation, Attica Rehabilitation Center, 19018 Magoula, Greece; eirinipavlou123@gmail.com; 4 Department of Informatics and Telecommunication, National and Kapodistrian University of Athens, 15784 Athens, Greece; eliasm@di.uoa.gr; 5 Department of Nutrition, Harokopeion University, 17676 Athens, Greece; 6 Department of Neurology, Columbia University Medical Center, New York, NY 10032, USA; 7 Department of Nutrition and Dietetics Sciences, School of Health Sciences, Hellenic Mediterranean University, 72100 Crete, Greece; 8 Clinical and Epidemiological Neuroscience (NeuroÈpia), Institut d’Investigació Sanitària Pere Virgili (IISPV), 43204 Reus, Spain

**Keywords:** objective sleep, longitudinal design, cognition, cognitive aging, mild cognitive impairment

## Abstract

Introduction: Sleep disturbances are common as we age and have been linked to poor cognition and increased cognitive decline. Objective: We aimed to examine cross-sectional and longitudinal associations between objective sleep measures and cognition in middle-aged and older adults, including cognitively healthy (CH) individuals and those with mild cognitive impairment (MCI). Methods: Participants from the Aiginition Longitudinal Biomarker Investigation Of Neurodegeneration (ALBION) study (age > 40) underwent 7-day wrist actigraphy (Actiwatch 2). Sleep exposures included sleep duration, sleep efficiency, sleep variability, sleep onset latency, wake after sleep onset (WASO), and number of awakenings. A neuropsychological battery was administered examining memory, executive function, visuospatial ability, language, attention speed, and a global composite score. Cross-sectional associations were tested using generalized linear models (adjusted for age, sex, education). Longitudinal associations with cognitive trajectories were examined with linear mixed-effect models. Results: In total (N = 184; 65% women; mean age 65 years), average sleep duration was 7.2 h and mean sleep efficiency was at 80%. Cross-sectionally, more nightly awakenings were associated with poor memory and attention speed. In a 1.5-year follow-up, (n = 93), higher baseline sleep efficiency was associated with better memory and language performance, while longer WASO, more awakenings, and longer sleep onset latency showed nominal associations with less favorable cognitive trajectories, although these associations did not remain statistically significant after FDR correction. Time-varying analyses indicated that sleep variability showed robust non-linear associations with poorer memory trajectories over follow-up and remained significant after FDR adjustment; significant mean change in awakenings and variability appeared to intensify in later follow-up phases. The association between sleep characteristics and cognitive decline varied across follow-up time, with stronger adverse changes observed during later follow-up phases. Discussion: Objective indicators of sleep continuity, especially sleep variability, were most consistently related to domain-specific cognitive outcomes, with strongest evidence for memory over time. Sleep fragmentation and irregular sleep patterns may represent potentially modifiable targets for future strategies aimed at preserving cognitive health during aging.

## 1. Introduction

Sleep is increasingly recognized as an important correlate of cognitive aging [1,2,3,4,5,6]. A growing body of evidence shows that specific sleep parameters, such as sleep duration, sleep efficiency, and sleep-disordered breathing, are cross-sectionally associated with poor cognitive outcomes in aging populations [7,8,9,10]. However, many previous studies were cross-sectional, relied on brief global screening instruments, or examined single sleep parameters in isolation. As a result, less is known about how multiple objectively measured sleep characteristics relate to domain-specific cognitive performance and longitudinal cognitive change across midlife and older age. Longitudinal studies examining whether sleep predicts cognitive changes over time in cognitively healthy older adults or in those with Mild Cognitive Impairment (MCI) are fewer but nevertheless informative. These studies collectively suggest that sleep is not only associated with cognition, cross-sectionally, but may also play a predictive role in the trajectory of cognitive aging. For instance, poorer sleep quality, reduced sleep efficiency, increased sleep fragmentation, and the presence of sleep-disordered breathing have all been linked to accelerated decline in cognitive domains such as memory, executive functioning, and processing speed [11,12,13]. Furthermore, individuals with persistent sleep disturbances appear more likely to convert from normal cognition to MCI, or from MCI to dementia, compared with those who maintain more stable sleep patterns [14,15,16]. Despite growing interest in sleep and cognitive aging, important gaps remain in the literature. Many studies have relied on cross-sectional designs, limiting inference about cognitive change over time, while others have used subjective sleep measures that are vulnerable to recall bias and measurement error. In addition, cognition has often been assessed using brief global screening instruments such as the Mini-Mental State Examination (MMSE) rather than comprehensive neuropsychological batteries capable of detecting domain-specific patterns [17,18]. Several studies using actigraphy in older adults without dementia have reported associations between lower sleep efficiency, greater sleep fragmentation, irregular sleep patterns, and poorer cognitive performance [19,20]. However, findings have been inconsistent, and relatively few studies have examined whether objectively measured sleep is associated with longitudinal change across specific cognitive domains. Beyond average sleep duration and sleep efficiency, increasing attention has focused on sleep regularity and night-to-night variability as potentially important determinants of cognitive aging. Greater sleep variability may reflect circadian dysregulation and instability in sleep–wake timing, which have been linked to impaired glymphatic clearance, neuroinflammatory processes, metabolic dysregulation, and hippocampal vulnerability [21,22,23]. These mechanisms may be particularly relevant for memory processes, given the central role of sleep continuity and circadian stability in supporting memory function [24]. Although emerging evidence suggests that sleep irregularity may contribute to adverse cognitive outcomes, relatively few studies have examined whether objectively measured sleep variability and other actigraphy-derived sleep characteristics are associated with longitudinal changes across specific cognitive domains [21]. Less is also known about these associations in samples spanning midlife through older age. Therefore, the present study aimed to examine cross-sectional and longitudinal associations between multiple actigraphy-derived sleep measures and domain-specific cognition in middle-aged and older adults.

## 2. Methods

### 2.1. Participants

Participants were drawn from the Aiginition Longitudinal Biomarker Investigation Of Neurodegeneration (ALBION) cohort. The ALBION cohort includes individuals aged over 40 who report memory concerns and are either cognitively healthy (CH) or have MCI [25,26]. ALBION is an ongoing longitudinal study addressing multiple research questions related to preclinical Alzheimer’s disease (AD) [25,26], aiming to identify potential markers for early diagnosis, prediction, and prevention [25,26]. The study collects comprehensive data, including demographic, social and medical information; detailed neuropsychological assessments; and both objective and subjective sleep measures. Participants are assessed annually [26]. Written informed consent was obtained from all participants. The study was approved by the Institutional Review Board of Aiginition hospital (#255/10 May 2021).

Cognitive status was determined by an experienced neurologist (NS) specializing in cognitive disorders, based on clinical information, reliable collateral history, and performance on a comprehensive neuropsychological battery. Dementia diagnoses were established according to DSM-IV-TR criteria [27], and probable or possible Alzheimer’s disease was diagnosed using NINCDS/ADRDA criteria [28]. MCI and MCI subtypes (memory, executive speed, visuospatial, language, and combinations) were assigned when participants reported subjective cognitive complaints and demonstrated objective impairment in at least one cognitive domain, while maintaining preserved activities of daily living. Additional details regarding the cohort design and diagnostic procedures have been previously published [25,26]. 

### 2.2. Study Sample

**Figure 1:**
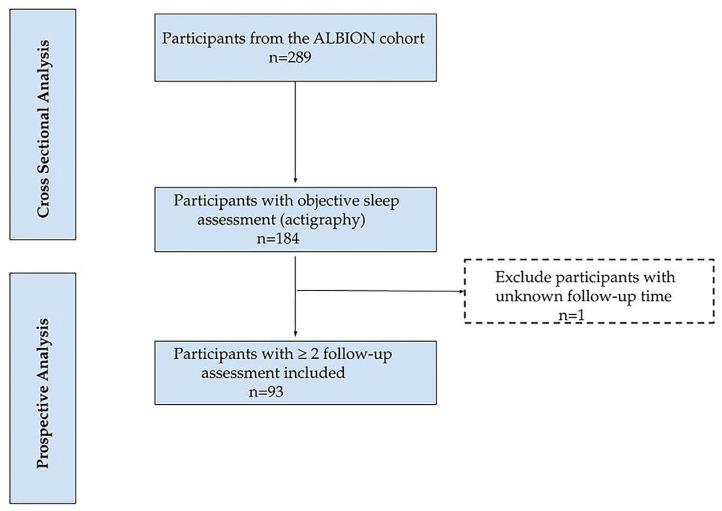
Flow chart of participants included in cross-sectional and prospective.

A total of 289 participants from the ALBION cohort were initially considered for inclusion. Of these, 184 participants had valid 7-day wrist actigraphy recordings and were included in the cross-sectional analyses. For prospective analyses, participants were required to have at least two cognitive follow-up assessments. After excluding one participant with unknown follow-up time, 93 participants met these criteria and were included in the longitudinal analyses. A flowchart summarizing participant inclusion and exclusion across analytical stages is presented in Figure 1.

To assess the possibility of selection bias, we compared baseline characteristics across three contrasts: (i) participants with MCI versus cognitively healthy individuals, (ii) participants with and without valid actigraphy data, and (iii) participants included versus not included in the longitudinal analyses. These comparisons examined sociodemographic factors (age, sex, years of education), MCI status, and baseline performance across cognitive domains.

### 2.3. Sleep Measures

Objective sleep–wake function was assessed using wrist-worn Actiwatch 2 devices (Philips Respironics, Murrysville, PA, USA) over a 7-day recording period. Actigraphy is a movement-based method that enables continuous monitoring of sleep–wake patterns in naturalistic settings [29]. Participants were instructed to wear the device continuously for seven consecutive days. For each sleep variable, mean values across recorded nights were calculated and used in the analyses.

The following actigraphy-derived sleep–wake parameters were examined: sleep duration (average hours slept per night), sleep variability (within-individual standard deviation of sleep duration across the recording period), sleep efficiency (total sleep time divided by time in bed × 100), sleep onset latency, WASO (defined as the total minutes scored as awake during sleep periods), and number of nightly awakenings (defined as the total number of wake bouts during sleep periods). These parameters reflect objective estimates of sleep quantity, continuity, and regularity [30]. 

### 2.4. Statistical Analysis

Baseline characteristics of participants were described as means and standard deviations (SD), or medians and interquartile ranges for quantitative variables, and percentages for categorical variables. These variables included demographic and sleep-related characteristics and cognitive scores.

Each of the above sleep measures was entered into the models separately as a main predictor. Where appropriate, variables were examined both as continuous and categorical measures. In particular, sleep efficiency was classified as suboptimal (<85%) or sufficient (≥85%), with sufficient (≥85%) set as the reference category [7]. Sleep duration was classified into three levels: short (<7 h), normal (7–9 h), or long (>9 h) [31,32]. To facilitate comparison with previous studies, we used normal (7–9 h) as the reference category. Participants were also categorized by median split for sleep duration variability [33], with low sleep variability (<1.24 h, corresponding to the median sleep variability in the sample) set as the reference category.

To account for potential confounding, covariate selection was informed by prior literature and a directed acyclic graph (DAG) framework developed using DAGitty v3.1 to identify factors potentially associated with both sleep characteristics and cognitive outcomes (Figure S1). Based on this framework, age (years), sex (men, women), and education (years) were included as covariates in all statistical models. Longitudinal analyses were additionally adjusted for diagnostic group (presence of MCI) (Supplementary Figure S1) [5,8,12,19]. This approach is consistent with previous research showing that these demographic factors are associated with both sleep parameters and cognitive performance in older adults [34].

The sample was determined by participant availability within the ALBION cohort and completeness of actigraphy and cognitive follow-up data. Model complexity was restricted by limiting covariate inclusion to variables identified a priori to reduce the risk of overfitting.

### 2.5. Missing Data

Prior to imputation, the extent of missingness was assessed, revealing a missingness rate of 1.09% for the variable of education. Aiming to maintain the largest possible sample size, we employed the random forest imputation method (“missForest” function from the “missForest” R package), a robust nonparametric approach that handles both numeric and categorical variables [35], for addressing missing variables despite the small proportion of missing data. All analyses were conducted using R (version 4.4.2; Team R 2025).

### 2.6. Cross-Sectional Analyses

Associations with cognitive outcomes were examined using Generalized Linear Models (GLMs) with appropriate distributions (Gaussian for z-scores, binomial for MCI status). Sleep duration, sleep variability, and sleep efficiency were analyzed both continuously and categorically. In addition, the remaining sleep predictors (sleep onset latency, wake after sleep onset, and number of awakenings) were separately introduced in the models as continuous variables. The association between sleep measures and cognitive function was estimated by beta coefficients and their 95% CIs obtained from generalized linear regression.

### 2.7. Prospective Analyses

Since participants contributed several cognitive assessments over the follow-up, the dataset had a longitudinal repeated-measures structure. Longitudinal models require at least two observations per participant. Individuals with only one study visit were excluded from the analysis. We also excluded participants who had missing cognitive score data or for whom the timing of follow-up assessments could not be determined.

For longitudinal analyses, linear mixed-effects models [12] were used to evaluate associations between baseline sleep metrics and cognitive trajectories during follow-up. These models incorporated all available cognitive assessments while accounting for within-person correlations over time and incomplete follow-up data. Participant identification numbers were used to define repeated observations within individuals, and both random intercepts and random slopes for time were included to account for interindividual baseline differences and variability in rates of cognitive change. Models included baseline sleep metrics, time (years since baseline), age, sex, education, and diagnostic group (presence of MCI). Sleep metrics were additionally modeled in interaction with time. Regression coefficients (β) represent expected changes in cognitive z-scores associated with one-unit differences in sleep parameters while accounting for longitudinal within-person change. Parameter estimates, 95% confidence intervals, and *p*-values were extracted from fitted models.

### 2.8. Exploratory Analyses of Non-Linear Sleep × Time Associations

To evaluate whether associations between sleep parameters and cognitive trajectories varied non-linearly over follow-up, exploratory linear mixed-effects models incorporating natural cubic splines (3 degrees of freedom) for time were fitted [12]. Models included interaction terms between each sleep exposure (treated as continuous) and spline-transformed time to assess potential non-linear sleep × time associations. Sleep exposures demonstrating evidence of non-linear interactions were further explored across empirically derived follow-up phases (early, middle, late) to facilitate interpretation of time-varying associations, and phase-specific estimates were derived to characterize how associations changed over follow-up.

To account for multiple testing in both cross-sectional and prospective analyses, *p*-values for the multivariable-adjusted associations were corrected using the Benjamini–Hochberg false discovery rate (FDR) procedure [36]. An FDR-adjusted *p*-value < 0.05 was considered statistically significant for the analyses of the six sleep metrics across the six cognitive domains [37], while a conventional *p*-value < 0.05 was used to define significance in all other analyses.

## 3. Results

### 3.1. Participants’ Characteristics

Among the 289 individuals initially assessed, 184 participants had valid wrist actigraphy data and were included in the present analyses (Table 1). Participants were predominantly women, with a mean age of 65.0 years. Mean educational attainment was 14.5 years (SD = 3.5), and the median follow-up duration was 1 year (IQR: 0–2). Overall, 47.8% of participants were classified as having MCI.

Data are presented as mean (SD) or as median (interquartile range) for continuous variables, and as n (%) for categorical variables. Data was missing and therefore sample and sizes were smaller for ZME (n = 181), ZEX (n = 178), ZVS (n = 181), ZLA (n = 180), ZAS (n = 180), ZCO (n = 181). Low * sleep variability indicates participants with variability less than 1.25 h, which corresponds to the median sleep variability in the sample. High * sleep variability indicates participants with variability greater than 1.25 h, which corresponds to the median sleep variability in the sample. Low ** sleep efficiency indicates participants with efficiency equal or less than 85%. High ** sleep efficiency indicates participants with efficiency more than 85%.

Average nightly sleep duration was 7.2 h (SD = 1.3), with 47.3% of participants sleeping 7–9 h, 44.0% sleeping less than 7 h, and 8.7% sleeping more than 9 h per night. Mean sleep variability was 1.47 h (SD = 0.86), with equal proportions classified as low and high variability. Mean sleep efficiency was 80.3% (SD = 9.2), with 69.6% of participants classified as having low sleep efficiency and 30.4% as having high sleep efficiency. Additional sleep parameters, including sleep onset latency, WASO, and number of nocturnal awakenings, are presented in Table 1.

Baseline characteristics stratified by cognitive status are presented in Table 2. Participants with MCI were older than cognitively healthy individuals (mean age 67.5 vs. 62.7 years, *p* < 0.001). Regarding sleep parameters, sleep efficiency was lower among participants with MCI compared to cognitively healthy individuals (*p* = 0.024). No statistically significant differences were observed for the remaining sleep parameters (all *p* > 0.05), nor for categorical sleep measures.

Baseline differences between CH and MCI were evaluated using independent samples *t*-tests or Mann–Whitney tests for continuous variables, depending on normality assumptions, and χ^2^ or Fisher’s exact tests for categorical variables. Low * sleep variability indicates participants with variability less than 1.25 h, which corresponds to the median sleep variability in the sample. High * sleep variability indicates participants with variability greater than 1.25 h, which corresponds to the median sleep variability in the sample. Low ** sleep efficiency indicates participants with efficiency equal or less than 85%. High ** sleep efficiency indicates participants with efficiency more than 85%.

As expected, participants with MCI performed significantly worse across all cognitive domains, including memory, executive function, visuospatial ability, language, attention speed, and global cognition (all *p* < 0.001). As shown in Supplementary Table S1, participants with and without valid actigraphy data did not differ in age, sex, or MCI prevalence. However, participants with actigraphy data had higher years of education and differed in selected cognitive domains, including memory (ZME), language (ZLA), and global cognition (ZCO).

As shown in Supplementary Table S2, participants included in the longitudinal analyses had fewer years of education and were more likely to be women (*p* = 0.045) compared to those not included. They also had a lower prevalence of MCI (33.0% vs. 63.3%) and showed better performance in memory, while no statistically significant differences were observed for other cognitive domains.

### 3.2. Cross-Sectional Associations Between Baseline Sleep Measures and Cognitive Outcomes

Results from generalized linear models showed that specific sleep parameters were significantly associated with cognitive performance after multivariable adjustment (Table 3 and Table 4). In particular, a higher number of nocturnal awakenings was associated with poor performance in selected cognitive domains. Specifically, each additional awakening was associated with low performance in the memory domain (ZME; β = −0.009, 95% CI: −0.02 to −0.00, *p* = 0.032) and the attention speed domain (ZAS; β = −0.010, 95% CI: −0.02 to −0.00, *p* = 0.018) (Table 3).

Regression analysis was used with multivariable-adjusted β-coefficients (95% CI). The models adjusted for sex, age (years), and education (years). All models were estimated using generalized linear models (GLMs) with Gaussian (identity) distribution for all outcomes. FDR-controlled adjustments were conducted by applying the method of Benjamini and Hochberg. FDR represents false discovery rate. * A two-sided *p*-value < 0.05 was considered significant.

**Table 4:** Cross-sectional associations between categorical sleep measures and cognitive domain test z-scores in total sample.

Outcome	Normal	Sleep Duration Short β (95% CI)	*p*-Value	FDR	Long β (95% CI)	*p*-Value	FDR
ZME (Memory)	Ref	0.09 (−0.23, 0.41)	0.579	0.897	−0.08 (−0.63, 0.48)	0.790	0.897
ZEX (Executive)	Ref	0.194 (−0.05, 0.44)	0.118	0.753	0.046 (−0.38, 0.47)	0.831	0.897
ZVS (Visuospatial)	Ref	0.219 (−0.61, 1.04)	0.603	0.897	0.372 (−1.04, 1.79)	0.606	0.897
ZLA (Language)	Ref	−0.031 (−0.35, 0.29)	0.852	0.897	−0.149 (−0.70, 0.40)	0.597	0.633
ZAS(Attention speed)	Ref	0.137 (−0.17, 0.44)	0.380	0.897	−0.409 (−0.93, 0.11)	0.125	0.753
ZCO (Composite)	Ref	0.239 (−0.19, 0.67)	0.274	0.897	0.049 (−0.68, 0.78)	0.897	0.897
	Low	Sleep Variability High * β (95% CI)	*p*-value	FDR			
ZME (Memory)	Ref	−0.138 (−0.44, 0.17)	0.376	0.638			
ZEX (Executive)	Ref	−0.073 (−0.30, 0.16)	0.535	0.642			
ZVS (Visuospatial)	Ref	−0.326 (−1.11, 0.47)	0.415	0.638			
ZLA (Language)	Ref	0.047 (−0.26, 0.35)	0.762	0.762			
ZAS(Attention speed)	Ref	−0.119 (−0.41, 0.17)	0.425	0.638			
ZCO (Composite)	Ref	−0.216 (−0.62, 0.19)	0.298	0.638			
	High *	Efficiency Low ** β (95% CI)	*p*-value	FDR			
ZME (Memory)	Ref	−0.220 (−0.55, 0.11)	0.192	0.576			
ZEX (Executive)	Ref	0.001 (−0.25, 0.25)	0.994	0.994			
ZVS (Visuospatial)	Ref	−0.101 (−0.95, 0.75)	0.817	0.994			
ZLA (Language)	Ref	−0.363 (−0.69, −0.04)	0.031 *	0.185			
ZAS (Attention speed)	Ref	0.026 (−0.29, 0.34)	0.871	0.994			
ZCO (Composite)	Ref	−0.169 (−0.61, 0.27)	0.455	0.909			

Regression analyses were performed using generalized linear models (GLMs) using multivariable-adjusted β-coefficients (95% CI). Model 1 adjusted for sex, age (years), and education (years). High * sleep variability indicates participants with variability more than 1.24 h, which corresponds to the median sleep variability in the sample. Low ** sleep efficiency indicates participants with efficiency equal or less than 85%. * A two-sided *p*-value < 0.05 was considered significant.

Higher sleep efficiency was associated with better language performance (ZLA) compared with low sleep efficiency. However, none of the cross-sectional associations remained statistically significant after correction for multiple testing.

### 3.3. Longitudinal Associations Between Sleep Measures and Cognitive Outcomes

Repeated cognitive assessments were available for 93 participants and were used to examine longitudinal changes in cognitive performance over time. During follow-up, several baseline sleep parameters were significantly associated with cognitive trajectories (Table 5 and Table 6). Specifically, higher baseline sleep efficiency was associated with better subsequent language performance over time and showed a nominal association with memory trajectories. In contrast, longer WASO and a greater number of nocturnal awakenings showed nominal associations with steeper decline in memory performance over follow-up; however, these associations did not remain statistically significant after FDR correction. Additionally, greater nocturnal awakenings and longer sleep onset latency showed nominal associations with less favorable language trajectories, while longer sleep onset latency also showed nominal associations with attention speed and global cognitive performance; these findings did not survive FDR correction.

**Table 6:** Longitudinal associations of categorical sleep measures with cognitive domain z-scores.

Outcome	Normal	Sleep Duration Short β (95% CI)	*p*-Value	FDR	Long β (95% CI)	*p*-Value	FDR
ZME (Memory)	Ref	−0.022 (−0.39, 0.35)	0.906	0.946	−0.533 (−1.14, 0.08)	0.085	0.409
ZEX (Executive)	Ref	0.062 (−0.27, 0.40)	0.712	0.876	0.132 (−0.41, 0.68)	0.633	0.876
ZVS (Visuospatial)	Ref	0.274 (−1.16, 1.71)	0.704	0.876	0.904 (−1.45, 3.26)	0.447	0.876
ZLA (Language)	Ref	−0.340 (−0.63, −0.05)	0.023 *	0.193	−0.255 (−0.73, 0.22)	0.291	0.876
ZAS (Attention speed)	Ref	−0.145 (−0.52,0.23)	0.441	0.876	0.045 (−0.56, 0.65)	0.833	0.946
ZCO (Composite)	Ref	0.108 (−0.61, 0.82)	0.766	0.897	0.246 (−0.93,1.42)	0.680	0.876
	Low	Sleep Variability High β (95% CI)	*p*-value	FDR			
ZME (Memory)	Ref	−0.268 (−0.63, 0.09)	0.141	0.561			
ZEX (Executive)	Ref	−0.213 (−0.53, 0.10)	0.187	0.561			
ZVS (Visuospatial)	Ref	−0.835 (−2.18, 0.51)	0.222	0.561			
ZLA (Language)	Ref	−0.009 (−0.30, 0.28)	0.949	0.949			
ZAS (Attention speed)	Ref	−0.240 (−0.60, 0.12)	0.184	0.561			
ZCO (Composite)	Ref	−0.546 (−1.21, 0.12)	0.107	0.561			
	High	Efficiency Low β (95% CI)	*p*-value	FDR			
ZME (Memory)	Ref	−0.172 (−0.56, 0.22)	0.381	0.894			
ZEX (Executive)	Ref	−0.038 (−0.38, 0.31)	0.828	0.894			
ZVS (Visuospatial)	Ref	−0.657 (−2.14, 0.82)	0.380	0.894			
ZLA (Language)	Ref	−0.284 (−0.59, 0.02)	0.070	0.894			
ZAS (Attention speed)	Ref	−0.035 (−0.42, 0.35)	0.856	0.894			
ZCO (Composite)	Ref	−0.349 (−1.08, 0.39)	0.348	0.894			

Linear mixed-effects models with random intercepts and slopes for time were used to assess longitudinal associations between sleep parameters and cognitive domain z-scores. Time was modeled using natural cubic splines with three degrees of freedom to account for non-linear trajectories over follow-up. Only main effects of sleep variables are presented here. Models were adjusted for age, sex, education, and mild cognitive impairment (MCI) status. Reported are estimated regression coefficients (β) and 95% confidence intervals (CIs). False discovery rate (FDR) correction was applied using the Benjamini–Hochberg method. * A two-sided *p*-value < 0.05 was considered significant.

Additionally, participants with a higher number of nocturnal awakenings and longer sleep onset latency exhibited a faster decline in language function. Prolonged sleep onset latency was also significantly associated with poorer trajectories in attention speed and global cognitive performance.

### 3.4. Time-Varying Associations Between Sleep and Cognition

Several sleep characteristics showed time-dependent associations with cognitive outcomes. In spline-based longitudinal models, a higher number of nocturnal awakenings was associated with lower subsequent memory performance (ZME), with the first spline component showing a small but statistically significant negative association. Sleep duration was associated with longitudinal language trajectories (ZLA), with longer baseline sleep duration predicting poorer subsequent language performance over time, as captured by a statistically significant second spline component; however, these effects did not survive FDR correction (Table 7).

We determined spline non-linear interaction effects between follow-up time and sleep exposures on cognitive outcomes. Estimates are adjusted for age, sex, education, and mild cognitive impairment (MCI) status. Reported are estimated regression coefficients (β) and 95% confidence intervals (CIs). False discovery rate (FDR) correction was applied using the Benjamini–Hochberg method. * A two-sided *p*-value < 0.05 was considered significant. Only exposures with significant non-linear interaction are shown.

**Figure 2:**
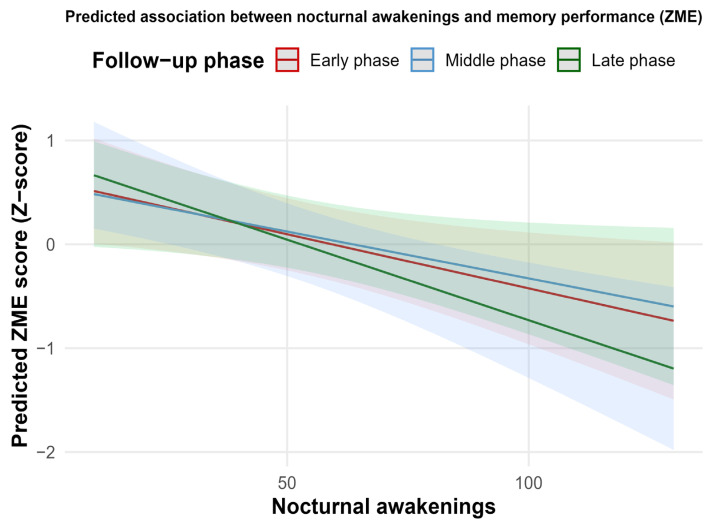
Predicted marginal effects from mixed-effects models showing the association between nocturnal awakenings and memory (ZME) across Early, Middle, and Late phases of follow-up. The association is consistently negative, with steeper declines observed in the late phase. Models were adjusted for age, sex, education, and MCI status. Shaded areas represent 95% confidence intervals.

By contrast, sleep variability demonstrated consistent and robust associations with future memory performance. Both the second and third spline components were statistically significant (β = −0.813 and β = −1.379, respectively; both *p* < 0.001) and remained significant after FDR adjustment (Table 7). After categorizing follow-up time into tertiles, three approximately equal-sized time phases were defined: Early (n = 93), Middle (n = 92), and Late (n = 92). Phase-specific trend estimates (Table 8) indicated that a higher number of nocturnal awakenings was associated with poorer memory performance across all phases, with the strongest and statistically significant association observed in the late phase (trend = −0.015; 95% CI: −0.02 to −0.006). These trajectories are illustrated in Figure 2 and Figures S2–S5.

Estimates are adjusted for age, sex, education, and mild cognitive impairment (MCI) status. Reported are estimated marginal trends derived from the fitted linear mixed-effects model using post-estimation procedures (emtrends), reflecting phase-specific sleep effects (β) and 95% confidence intervals (CIs). * A two-sided *p*-value < 0.05 was considered statistically significant. Only exposures with significant non-linear sleep × time interactions are presented. In Supplementary Tables S3–S8 we present all data including the non-significant associations.

**Figure 3:**
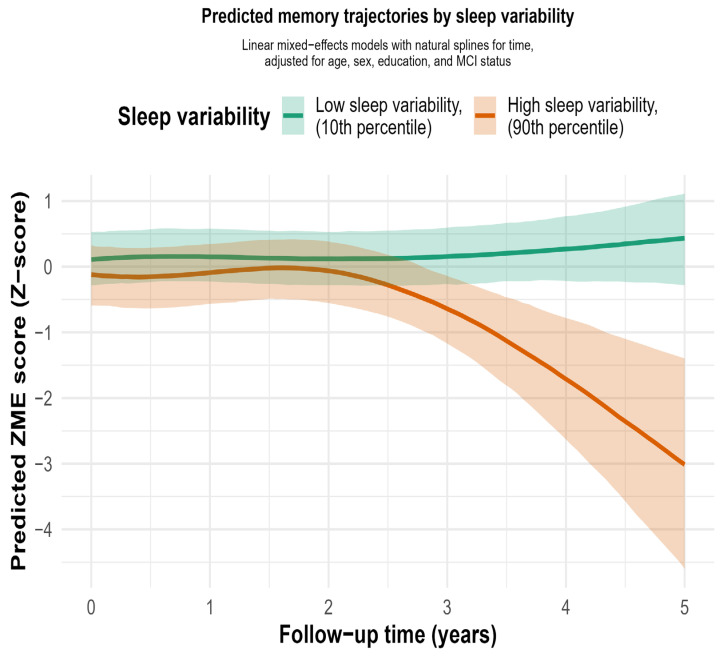
Predicted memory trajectories (ZME) over follow-up for participants with high (90th percentile) versus low (10th percentile) sleep variability, based on mixed-effects models adjusted for age, sex, education, and MCI status. Shaded areas represent 95% confidence intervals.

Similarly, higher sleep variability was progressively more strongly associated with poorer memory performance over follow-up. Associations were negative but non-significant in the early and middle phases, whereas in the late phase the association reached statistical significance (trend = −0.235; 95% CI: −0.45 to −0.02) (Table 8; Figure 3). No statistically significant phase-specific associations were observed between sleep duration and language performance.

## 4. Discussion

In this study we leveraged objective, multi-night sleep assessment and a domain-specific neuropsychological battery to clarify how sleep relates to cognitive function in midlife and older age within the ALBION cohort. Cross-sectionally, most sleep metrics showed weak or null associations with cognitive performance after correction for multiple testing, consistent with the notion that single-time-point sleep assessments may be insufficient to capture the cumulative or dynamic influence of sleep on brain health. Nevertheless, markers of sleep fragmentation were associated with poor cognitive performance, with a higher number of nocturnal awakenings related to poor memory and attention speed. Importantly, these modest cross-sectional findings contrasted with clearer patterns observed in longitudinal analyses, underscoring the added value of repeated cognitive assessment for identifying sleep-related risk factors [38]. A key contribution of the present study lies in its longitudinal analyses, which provide insight beyond cross-sectional snapshots by capturing within-person cognitive change over time. These analyses included both cognitively healthy individuals and participants with mild cognitive impairment at baseline and were conducted in the combined sample, with baseline cognitive status included as a covariate. Within this framework, several baseline sleep characteristics, particularly those reflecting sleep continuity and regularity, were associated with subsequent cognitive trajectories, most consistently in the memory domain. Higher sleep efficiency was associated with more favorable language trajectories and showed nominal associations with memory performance. Similarly, longer wake after sleep onset, greater nocturnal awakenings, and longer sleep onset latency demonstrated nominal longitudinal associations with less favorable cognitive trajectories across memory, language, attention speed, and global cognition; however, these findings did not remain statistically significant after correction for multiple testing. Nevertheless, the consistent directionality of these associations across related sleep metrics, cognitive domains, and modeling approaches may suggest the presence of an underlying longitudinal signal rather than isolated chance findings [18,34].

Importantly, the strongest and most robust longitudinal finding concerned sleep variability. Greater night-to-night variability in sleep duration showed non-linear associations with worsening memory over follow-up and remained statistically significant after correction for multiple testing. Time-varying and phase-specific analyses further indicated that the adverse impact of sleep irregularity on memory became more apparent with longer follow-up. This pattern is consistent with a cumulative process, whereby sustained sleep irregularity gradually affects cognitive outcomes, rather than reflecting an acute or stage-specific effect. These findings extend prior work suggesting that irregular sleep patterns may influence cognitive aging through multiple interconnected biological pathways. Greater night-to-night sleep variability may reflect circadian rhythm instability, which has been associated with impaired synchronization of neural and metabolic processes supporting cognition [39]. Experimental and epidemiological evidence further suggest that fragmented or irregular sleep may contribute to impaired glymphatic clearance, increased neuroinflammatory signaling, and metabolic dysregulation, mechanisms increasingly implicated in hippocampal dysfunction and memory decline [23,24,25,26,27,28,29,30,31,32,35,36,37,38,40]. Given the role of stable sleep architecture in supporting memory processes, these pathways may help explain why sleep variability showed the strongest and most consistent association with memory trajectories in our study. 

Overall, the present findings align with and extend previous longitudinal studies reporting associations between sleep fragmentation, poor sleep continuity, and accelerated cognitive decline, particularly in memory-related domains [40,41]. Prior studies using both subjective and objective sleep measures have similarly emphasized the importance of sleep regularity and continuity over average sleep duration and have suggested that fragmented or irregular sleep may precede and predict cognitive deterioration [42]. Notably, the inclusion of participants spanning midlife through older age allows the present study to capture sleep–cognition associations at earlier stages of cognitive aging, complementing prior work that has focused primarily on older populations [43]. Within this context, the current study adds novel evidence from objective, multi-night actigraphy and domain-specific cognitive trajectories in a well-characterized cohort.

Strengths of this study include objective sleep assessment over multiple nights, adjustment for relevant covariates, and the use of mixed-effects models that leveraged repeated cognitive assessments. In addition, correction for multiple testing was applied to reduce the likelihood of false-positive findings.

Several limitations should also be acknowledged. The longitudinal analytic sample was modest, which may have limited statistical power to detect smaller effects, particularly after correction for multiple testing, and constrained our ability to examine heterogeneity by cognitive status or other potential modifiers. The prospective analyses included a subset of participants with repeated cognitive assessments (n = 93), and participants retained longitudinally differed from those not included, including lower MCI prevalence and better baseline memory performance. This introduces the possibility of selection bias and may limit the generalizability of longitudinal findings. It is possible that participants retained over follow-up represent a healthier subgroup, potentially underestimating sleep–cognition associations observed in broader aging populations. Although actigraphy provides valid estimates of habitual sleep, it cannot fully characterize sleep architecture compared with polysomnography and may misclassify quiet wakefulness as sleep. Although models were adjusted for a range of relevant demographic and clinical covariates, residual confounding due to unmeasured or imperfectly measured factors cannot be fully excluded, as in all observational studies. Finally, follow-up duration was relatively short for some participants, which may have constrained the detection of longer-latency cognitive changes.

In summary, the present findings highlight sleep fragmentation and night-to-night irregularity as features that are consistently linked to memory-related cognitive aging. Across multiple longitudinal analyses, disrupted and irregular sleep showed consistent associations with memory trajectories, suggesting that these sleep characteristics may capture an underlying vulnerability relevant to cognitive decline. At the same time, the modest effect sizes and the attenuation of some associations after correction for multiple testing indicate that these results should be interpreted cautiously. Taken together, the findings should be viewed as hypothesis-generating and underscore the need for larger longitudinal studies with repeated sleep, cognitive, and biomarker assessments to clarify underlying mechanisms and to determine whether interventions aimed at improving sleep continuity and regularity may help preserve cognitive function with aging.

## Figures and Tables

**Table 1:** Characteristics of study participants in the total sample.

Sample Characteristics	N = 184
Age in years, mean (SD)	65 (9.5)
Education in years, mean (SD)	14.5 (3.5)
Sex, %	
Male	64 (34.8)
Female	120 (65.2)
Mild cognitive impairment, %	
No	96 (52.2)
Yes	88 (47.8)
**Sleep parameters (continuous)**	
Sleep duration (h/night), mean (SD)	7.22 (1.29)
Sleep variability (hours/night), mean (SD)	1.25 (0.92–1.77)
Efficiency (%)	83.9 (77.4–88.5)
Sleep onset latency (minutes/day)	23.5 (6.2–51.6)
WASO (minutes/day)	35.3 (22.9–51.0)
Number Awakenings	42 (31–59)
Sleep duration (h/night), n [%]	
<7	81 (44.02)
7–9	87 (47.28)
≥9	16 (8.70)
Sleep Variability (h/night), n [%]	
Low *	92 (50.0)
High *	92 (50.0)
Efficiency, n [%]	
Low **	128 (69.56)
High **	56 (30.43)
**Cognitive scores** (z-score)	
ZME (Memory)	−0.23 (−1.13–0.45)
ZEX (Executive function)	−0.13 (−0.66–0.34)
ZVS (Visuospatial)	0.02 (−0.62–0.65)
ZLA (Language)	0.06 (−0.51–0.43)
ZAS (Attention speed)	−0.02 (−0.63–0.50)
ZCO (Composite)	−0.11 (−0.58–0.30)

**Table 2:** Baseline characteristics of participants stratified by cognitive status (Cognitively Healthy vs. Mild Cognitive Impairment.

Sample Characteristics	Cognitive Healthy (CH) N = 96	Mild Cognitive Impairment (MCI) N = 88	*p*-Value
Age in years, mean (SD)	62.66 (8.87)	67.5 (9.58)	<0.001
Education in years, mean (SD)	14.74 (3.32)	14.26 (3.62)	0.351
Sex, %			0.370
Men	30 (31.3%)	34 (38.6%)	
Women	66 (68.8%)	54 (61.4%)	
**Sleep parameters (continuous)**			
Sleep duration (h/night), mean (SD)	7.18 (1.24)	7.25 (1.34)	0.737
Sleep variability (hours/night), mean (SD)	1.20 (0.88–1.77)	1.31 (0.98–1.78)	0.465
Efficiency (%)	82.8 (78.7–86.7)	81.6 (74.2–84.1)	0.024
Onset (minutes/day)	33.9 (18.9–53.1)	35.7 (22.0–63.6)	0.198
WASO (minutes/day)	36.3 (27.1–50.6)	42.4 (30.1–55.7)	0.061
Number Awakenings	43.3 (33.5–54)	45.1 (35.4–60.4)	0.195
Sleep duration (h/night), n [%]			0.895
<7	43 (44.8)	38 (43.2)	
7–9	44 (45.8)	43 (48.9)	
≥9	9 (9.4)	7 (8.0)	
Sleep Variability (h/night), n [%]			0.658
Low *	50 (52.1)	42 (47.7)	
High *	46 (47.9)	46 (52.3)	
Efficiency, n [%]			0.09
Low **	61 (63.5)	21 (23.9)	
High **	35 (36.5)	67 (76.1)	
**Cognitive scores** (z-score)			
ZME (Memory)	0.28 (−0.19–0.75)	−1.13 (−1.77–−0.45)	<0.001
ZEX (Executive function)	0.15 (−0.21–0.70)	−0.56 (−1.08–−0.11)	<0.001
ZVS (Visuospatial)	0.17 (−0.29–0.65)	−0.37 (−0.95–0.37)	<0.001
ZLA (Language)	0.25 (−0.11–0.59)	−0.36 (−1.14–0.08)	<0.001
ZAS (Attention speed)	0.23 (−0.32–0.77)	−0.47 (−1.06–0.11)	<0.001
ZCO (Composite)	0.27 (−0.09–0.45)	−0.55 (−1.28–0.17)	<0.001

**Table 3:** Cross-sectional associations between continuous sleep measures and cognitive domain test z-scores in total sample.

Outcome	Sleep Duration β (95% CI)	*p*-Value	FDR
ZME (Memory)	−0.069 (−0.19, 0.05)	0.257	0.533
ZEX (Executive)	−0.043 (−0.13, 0.05)	0.355	0.533
ZVS (Visuospatial)	−0.070 (−0.38, 0.24)	0.657	0.788
ZLA (Language)	−0.008 (−0.13, 0.11)	0.902	0.902
ZAS (Attention speed)	−0.093 (−0.21, 0.02)	0.111	0.533
ZCO (Composite)	−0.078 (−0.24, 0.08)	0.335	0.533
	Sleep Variability β (95% CI)	*p*-value	FDR
ZME (Memory)	−0.084 (−0.26, 0.09)	0.347	0.740
ZEX (Executive)	−0.016 (−0.15, 0.12)	0.816	0.816
ZVS (Visuospatial)	−0.149 (−0.60, 0.30)	0.520	0.779
ZLA (Language)	0.028 (−0.15, 0.20)	0.753	0.816
ZAS (Attention speed)	−0.081(−0.25, 0.09)	0.346	0.740
ZCO (Composite)	−0.107 (−0.34, 0.13)	0.370	0.740
	Efficiency β (95% CI)	*p*-value	FDR
ZME (Memory)	0.012 (−0.00, 0.03)	0.151	0.903
ZEX (Executive)	0.006 (−0.01, 0.02)	0.358	0.916
ZVS (Visuospatial)	−0.002 (−0.05, 0.04)	0.916	0.916
ZLA (Language)	0.003 (−0.01, 0.02)	0.731	0.916
ZAS (Attention speed)	0.004 (−0.01, 0.02)	0.597	0.916
ZCO (Composite)	0.002 (−0.02, 0.02)	0.832	0.916
	WASO β (95% CI)	*p*-value	FDR
ZME (Memory)	−0.007 (−0.01, 0.00)	0.080	0.482
ZEX (Executive)	−0.001 (−0.01, 0.00)	0.612	0.735
ZVS (Visuospatial)	0.011 (−0.01, 0.03)	0.253	0.507
ZLA (Language)	−0.001 (−0.01, 0.01)	0.848	0.848
ZAS (Attention speed)	−0.005 (−0.01, 0.00)	0.214	0.507
ZCO (Composite)	0.004 (−0.01, 0.01)	0.497	0.735
	Number of Awakenings β (95% CI)	*p*-value	FDR
ZME (Memory)	−0.009 (−0.02, −0.00)	0.032 *	0.095
ZEX (Executive)	−0.004 (−0.01, 0.00)	0.198	0.395
ZVS (Visuospatial)	−0.003 (−0.02, 0.02)	0.813	0.813
ZLA (Language)	−0.005 (−0.01, 0.00)	0.279	0.419
ZAS (Attention speed)	−0.010 (−0.02, −0.00)	0.018 *	0.095
ZCO (Composite)	−0.002 (−0.01, 0.01)	0.708	0.813
	Onset β (95% CI)	*p*-value	FDR
ZME (Memory)	−0.001 (−0.00, 0.00)	0.568	0.681
ZEX (Executive)	−0.001 (−0.00, 0.00)	0.385	0.577
ZVS (Visuospatial)	−0.005 (−0.01, 0.01)	0.350	0.577
ZLA (Language)	0.001 (−0.00, 0.00)	0.768	0.768
ZAS (Attention speed)	−0.002 (−0.01, 0.00)	0.248	0.577
ZCO (Composite)	−0.003 (−0.01, 0.00)	0.222	0.577

**Table 5:** Longitudinal associations of continuous sleep measures with cognitive domain z-scores.

Outcome	Sleep Duration β (95% CI)	*p*-Value	FDR
ZME (Memory)	−0.109 (−0.27, 0.05)	0.168	0.987
ZEX (Executive)	−0.001 (−0.14, 0.14)	0.987	0.987
ZVS (Visuospatial)	0.061 (−0.53, 0.66)	0.840	0.987
ZLA (Language)	0.058 (−0.07, 0.18)	0.363	0.987
ZAS (Attention speed)	0.045 (−0.11, 0.20)	0.567	0.987
ZCO (Composite)	0.007 (−0.29, 0.30)	0.960	0.987
	Sleep Variability β (95% CI)	*p*-value	FDR
ZME (Memory)	−0.101 (−0.30, 0.10)	0.323	0.597
ZEX (Executive)	−0.146 (−0.32, 0.03)	0.104	0.358
ZVS (Visuospatial)	−0.275 (−1.03, 0.48)	0.472	0.768
ZLA (Language)	−0.050 (−0.21, 0.11)	0.542	0.768
ZAS (Attention speed)	−0.119 (−0.32, 0.08)	0.237	0.512
ZCO (Composite)	−0.223 (−0.60, 0.15)	0.239	0.512
	Efficiency β (95% CI)	*p*-value	FDR
ZME (Memory)	0.031 (0.00, 0.06)	0.040 *	0.476
ZEX (Executive)	0.019 (−0.01, 0.05)	0.153	0.736
ZVS (Visuospatial)	0.047 (−0.07, 0.16)	0.411	0.926
ZLA (Language)	0.037 (0.01, 0.06)	0.002 *	0.051
ZAS (Attention speed)	0.026 (−0.00, 0.06)	0.078	0.621
ZCO (Composite)	0.029 (−0.03, 0.09)	0.310	0.926
	WASO β (95% CI)	*p*-value	FDR
ZME (Memory)	−0.010 (−0.02, −0.00)	0.020 *	0.473
ZEX (Executive)	−0.002 (−0.01, 0.01)	0.642	0.855
ZVS (Visuospatial)	0.019 (−0.01, 0.05)	0.269	0.707
ZLA (Language)	−0.005 (−0.01, 0.00)	0.189	0.707
ZAS (Attention speed)	−0.002 (−0.01, 0.01)	0.593	0.838
ZCO (Composite)	0.007 (−0.01, 0.02)	0.424	0.707
	Number of Awakenings β (95% CI)	*p*-value	FDR
ZME (Memory)	−0.011 (−0.02, −0.00)	0.014 *	0.262
ZEX (Executive)	−0.005 (−0.01, 0.00)	0.213	0.920
ZVS (Visuospatial)	−0.004 (−0.04,0.03)	0.808	0.920
ZLA (Language)	−0.008 (−0.01, −0.00)	0.022 *	0.262
ZAS (Attention speed)	−0.008 (−0.02, 0.00)	0.073	0.439
ZCO (Composite)	−0.003 (−0.02, 0.01)	0.747	0.920
	Onset β (95% CI)	*p*-value	FDR
ZME (Memory)	−0.005 (−0.01, 0.00)	0.089	0.335
ZEX (Executive)	−0.004 (−0.01, 0.00)	0.098	0.335
ZVS (Visuospatial)	−0.020 (−0.04, 0.00)	0.065	0.318
ZLA (Language)	−0.005 (−0.01, −0.00)	0.028 *	0.220
ZAS (Attention speed)	−0.006 (−0.01, −0.00)	0.027 *	0.220
ZCO (Composite)	−0.012 (−0.02, −0.00)	0.025 *	0.220

**Table 7:** Sleep × time spline interaction *p*-values for longitudinal cognitive outcome.

Sleep Measure	Cognitive Outcome	Spline Component	β (95% CI)	*p*-Value	FDR
Number of Awakenings	ZME (Memory)	Spline 1	−0.016 (−0.03, −0.00)	0.047	0.282
		Spline 2	0.002 (−0.02, 0.02)	0.844	0.951
		Spline 3	0.000 (−0.02, 0.02)	0.988	0.988
Sleep Duration	ZLA (Language)	Spline 1	0.126 (−0.15, 0.40)	0.373	0.831
		Spline 2	−0.321 (−0.63, −0.01)	0.041	0.243
		Spline 3	−0.330 (−0.76, 0.10)	0.127	0.762
Sleep Variability	ZME (Memory)	Spline 1	0.351 (−0.02, 0.72)	0.061	0.194
		Spline 2	−0.813 (−1.29, −0.33)	0.000 *	0.006
		Spline 3	−1.379 (−2.11, −0.64)	0.000 *	0.002

**Table 8:** Phase-specific trends between sleep measures and cognitive outcomes derived from linear mixed-effects models.

Sleep Measure	Cognitive Outcome	Time Phase	β (95% CI)	*p*-Value
Number of Awakenings	ZME (Memory)	Early	−0.010 (−0.02, −0.00)	0.021
		Middle	−0.009 (−0.02, −0.00)	0.046
		Late	−0.015 (−0.02, −0.01)	0.000
Sleep Duration	ZLA (Language)	Early	0.058 (−0.06, 0.18)	0.349
		Middle	−0.029 (−0.15, 0.09)	0.643
		Late	0.008 (−0.11, 0.13)	0.892
Sleep Variability	ZME (Memory)	Early	−0.117 (−0.33, 0.09)	0.271
		Middle	−0.131 (−0.34, 0.08)	0.224
		Late	−0.235 (−0.45, −0.02)	0.030 *

## Data Availability

The original contributions presented in this study are included in the article/Supplementary Material. Further inquiries can be directed to the corresponding author.

## Supplementary Materials

The following supporting information can be downloaded at https://www.mdpi.com/article/10.3390/medsci14030340/s1. Figure S1. Directed acyclic graph illustrating the hypothesized rela-tionships between objective sleep characteristics, cognitive outcomes, and covariates considered for confounder adjustment; Figure S2. Predicted memory trajectories (ZME) over follow-up for participants with a high (90th percentile) versus low (10th percentile) number of nightly awakenings, based on mixed-effects models adjusted for age, sex, education, and MCI status; Figure S3. Predicted marginal effects from mixed-effects models illustrating phase-specific associations between sleep duration and language performance (ZLA) across Early, Middle, and Late follow-up phases; Figure S4. Predicted trajectories of language performance (ZLA) for participants with long sleep duration (90th percentile) versus short sleep duration (10th percentile), based on mixed-effects models adjusted for age, sex, education, and MCI status; Figure S5. Predicted marginal effects from mixed-effects models showing the association between sleep variability and memory (ZME) across Early, Middle, and Late follow-up phases; Table S1. Baseline characteristics of ALBION participants with valid actigraphy data vs participants without actigraphy data; Table S2. Baseline characteristics of participants with actigraphy data included and not included in longitudinal analyses; Table S3. Cross-sectional associations between continuous sleep measures and cognitive domain z-scores in the total sample; Table S4. Cross-sectional associations between categorical sleep measures and cognitive domain test z-scores in total sample; Table S5. Longitudinal associations of continuous sleep measures with cognitive domain z-scores; Table S6. Longitudinal associations of categorical sleep measures with cognitive domain z-scores; Τable S7. Sleep × time spline interaction p-values for longitudinal cognitive outcome; Τable S8. Phase-specific trends between sleep measures and cognitive outcomes derived from linear mixed-effects models.
