# Supplementary material for: Objective Sleep Measures and Cognition in Middle-Aged and Older Adults: A Cross-Sectional and Longitudinal Analysis in the ALBION Study
Source: Med Sci (Basel). 2026 Jun 23;14(3):340. doi: 10.3390/medsci14030340 (PMC13413703; doi:10.3390/medsci14030340)
Supplement: Supplementary file 1 [file medsci-14-00340-s001.zip › medsci-4340461-supplementary.pdf]

Supplementary material

Supplementary figures

Supplementary Figure S1. Directed acyclic graph illustrating the hypothesized relationships between objective sleep characteristics, cognitive outcomes, and covariates considered for confounder adjustment. Age, sex, education, and MCI status were identified as factors potentially associated with both sleep characteristics and cognitive outcomes.

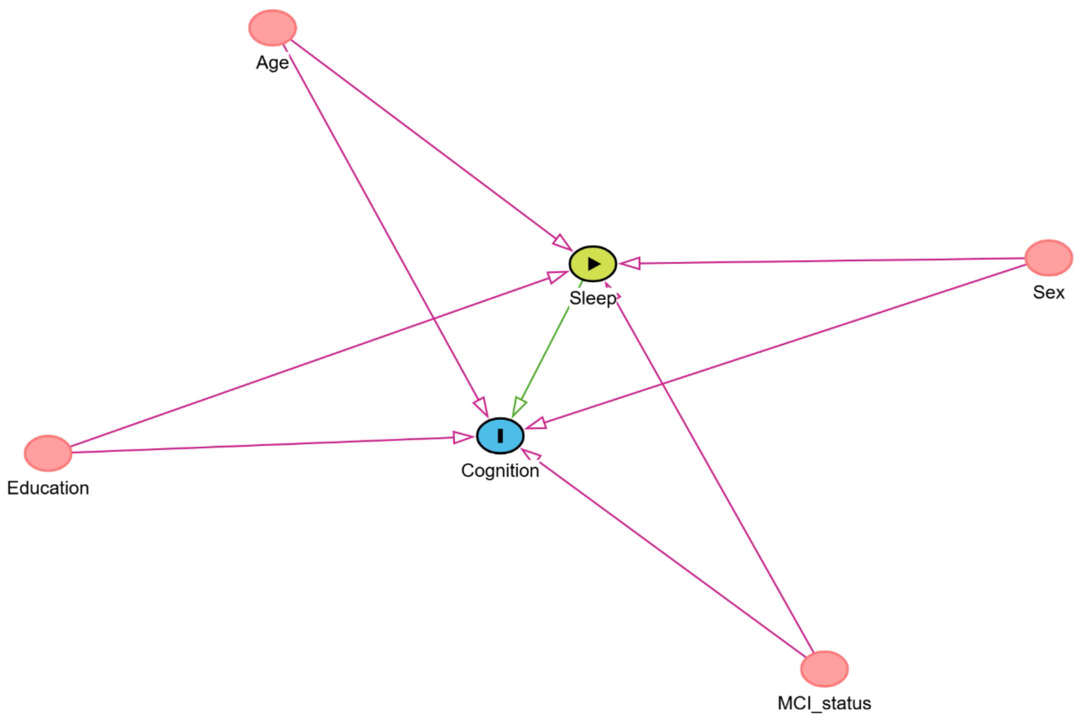

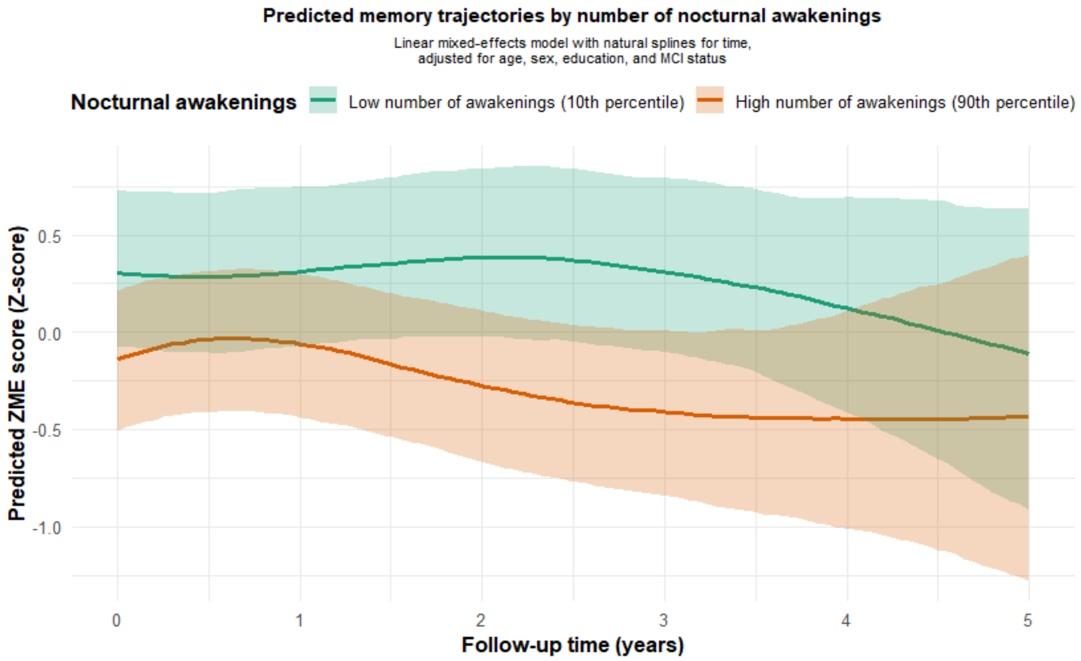

Supplementary Figure S2. Predicted memory trajectories (ZME) over follow-up for participants with a high (90th percentile) versus low (10th percentile) number of nightly awakenings, based on mixed-effects models adjusted for age, sex, education, and MCI status. Shaded areas represent 95% confidence intervals.

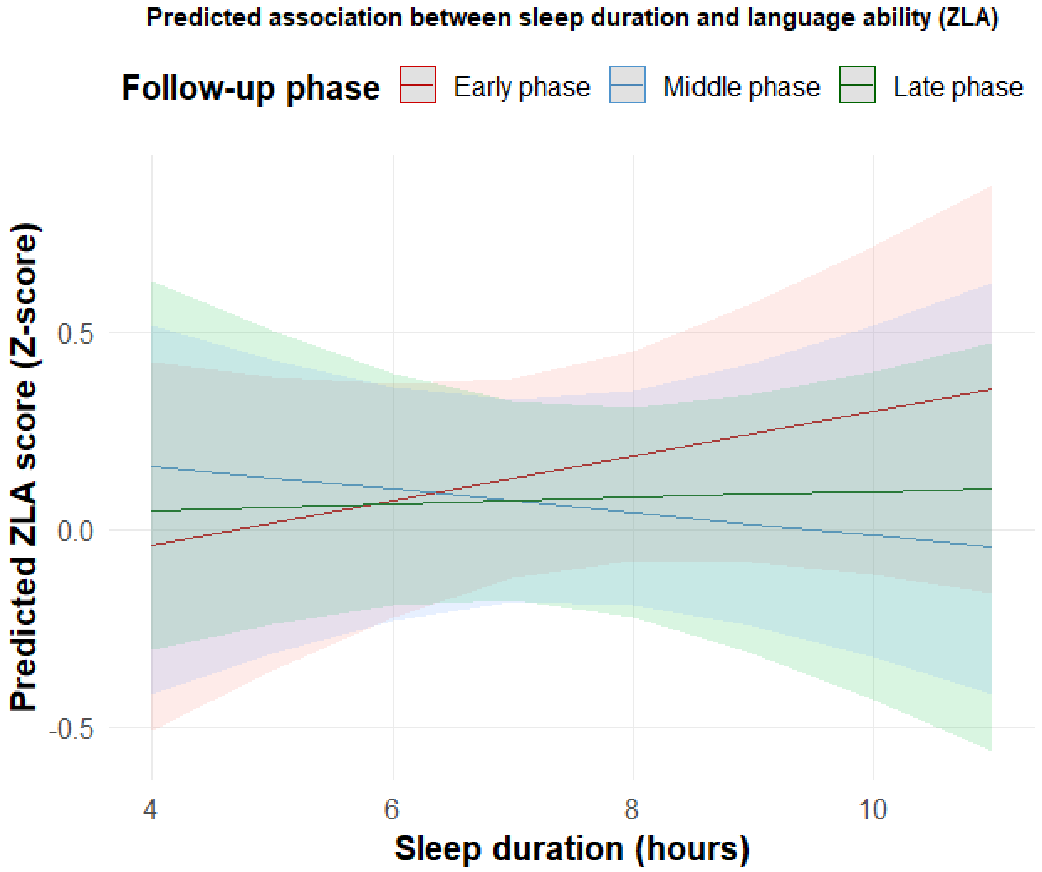

Supplementary Figure S3. Predicted marginal effects from mixed-effects models illustrating phase-specific associations between sleep duration and language performance (ZLA) across Early, Middle, and Late follow-up phases. Models were adjusted for age, sex, education, and MCI status. Shaded areas represent 95% confidence intervals.

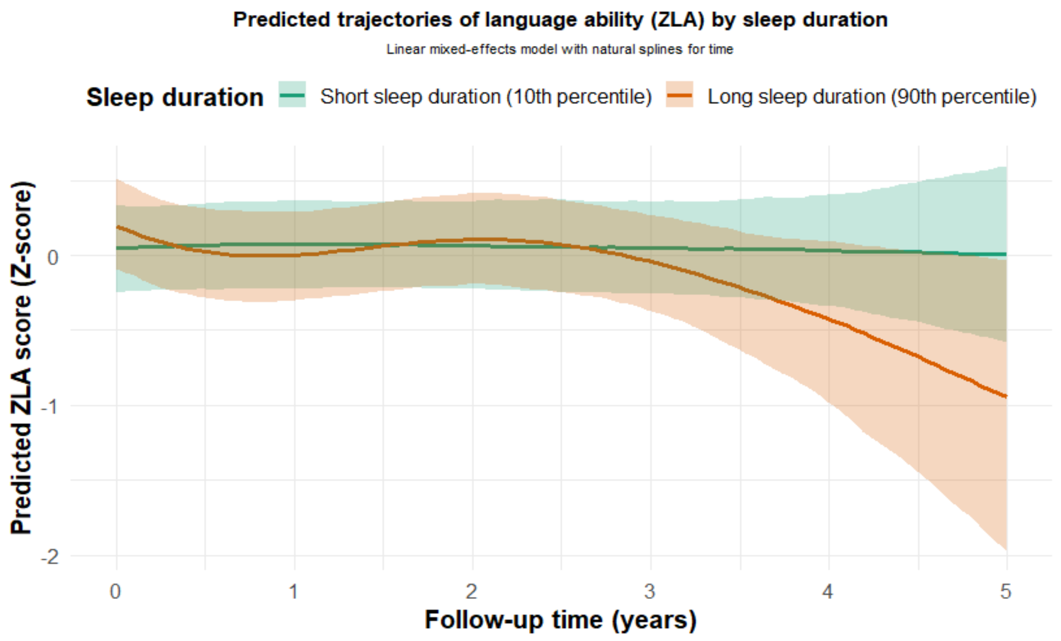

Supplementary Figure S4. Predicted trajectories of language performance (ZLA) for participants with long sleep duration (90th percentile) versus short sleep duration (10th percentile), based on mixed-effects models adjusted for age, sex, education, and MCI status. Shaded areas represent 95% confidence intervals.

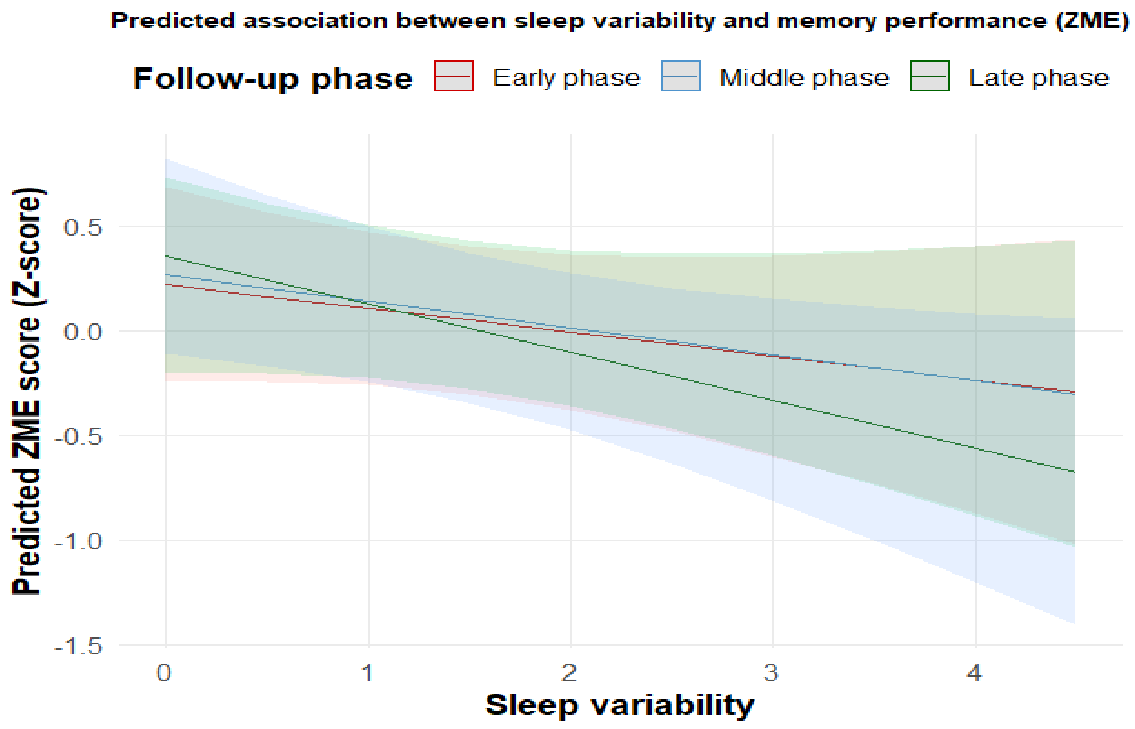

Supplementary Figure S5. Predicted marginal effects from mixed-effects models showing the association between sleep variability and memory (ZME) across Early, Middle, and Late follow-up phases. A progressively stronger negative association is observed in later phases. Models were adjusted for age, sex, education, and MCI status. Shaded areas represent 95% confidence intervals.

Supplementary Tables

Supplementary Table S1. Baseline characteristics of ALBION participants with valid actigraphy data vs participants without actigraphy data.

**Sample Characteristics**	ALBION cohort N=289	With actigraphy N = 184	Without actigraphy N = 105	P-value
Age in years, mean (SD)	65.2 (9.6)	65.0 (9.5)	65.4 (9.9)	0.737
Education in years, mean (SD)	14.2 (3.6)	14.5 (3.5)	13.5 (3.9)	0.028
Sex, female, n (%)	177 (61.2)	120 (65.2)	57 (54.3)	0.106
Mild cognitive impairment , Yes n (%)	146 (50.5)	88 (47.8)	58 (55.2)	0.241
**Cognitive scores** (z-score)				
ZME (Memory)	-0.47 (-1.42 - 0.32)	-0.23 (-1.13 - 0.45)	-0.69 (-1.90 - 0.01)	<0.001*
ZEX (Executive function)				0.073
ZVS (Visuospatial)	-0.18 (-0.86 - 0.33)	-0.13 (-0.66 - 0.34)	-0.39 (-1.19 - 0.32)	0.751
ZLA (Language)				<0.001*
ZAS (Attention-speed)	0.02 (-0.65 - 0.65)	0.02 (-0.62 - 0.65)	0.15 (-0.67 - 0.65)	0.184
ZCO (Composite) OK	-0.21 (-0.90 - 0.38)	0.06 (-0.51 - 0.43)	-0.52 (-1.32 - 0.11)	0.003*

	-0.09 (-0.65 - 0.47)	-0.02 (-0.63 - 0.50)	-0.16 (-0.87 - 0.35)
	-0.20 (-1.02 - 0.26)	-0.11 (-0.58 - 0.30)	-0.54 (-1.30 - 0.13)

Data are presented as mean (SD) or as median (interquartile range) for continuous variables and n (%) for categorical variables. Baseline differences between ALBION cohort participants who received actigraphy and those who did not, were evaluated using independent samples *t*-tests or Mann–Whitney tests for continuous variables, depending on normality assumptions, and χ^2 tests for categorical variables. *Statistical significance was set at *P* < 0.05.

Supplementary Table S2. Baseline characteristics of participants with actigraphy data included and not included in longitudinal analyses

**Sample Characteristics**	**Not Included**	**Included**	**P-value**
	**N =90**	**N=94**	
Age in years, mean (SD)	66.2 (9.7)	63.8 (9.3)	0.087
Education in years, mean (SD)	15.5 (2.8)	13.6 (3.8)	**<0.001***
Sex, female, n (%)	52 (57.8)	68 (72.3)	**0.045***
Mild cognitive impairment ,Yes n (%)	57 (63.3)	31 (33.0)	**<0.001***
**Cognitive scores** (z-score)			
ZME (Memory)	-0.74 (-1.32 - 0.20)	-0.02 (-0.74 - 0.53)	**0.006***
ZEX (Executive function)		-0.06 (-0.55 - 0.50)	0.081
ZVS (Visuospatial)	-0.30 (-0.68 - 0.18)	0.02 (-0.67 - 0.65)	0.967
ZLA (Language)		0.07 (-0.40 - 0.44)	0.128
ZAS (Attention-speed)	0.02 (-0.39 - 0.65)	-0.14 (-0.64 - 0.51)	0.477
ZCO (Composite)	-0.19 (-0.67 - 0.38)	-0.02 (-0.45 - 0.37)	0.062

	0.04 (-0.62 - 0.49)	
	-0.21 (-0.60 - 0.14)	

Data are presented as mean (SD) or as median (interquartile range) for continuous variables and n (%) for categorical variables. Differences in baseline characteristics between participants included in longitudinal analyses and those included in cross-sectional analyses only were evaluated using independent samples *t*-tests or Mann–Whitney tests for continuous variables, depending on normality assumptions, and χ^2 tests for categorical variables. *Statistical significance was set at P-value < 0.05.

Supplementary Table S3. Cross-sectional associations between continuous sleep measures and cognitive domain z-scores in the total sample.

Outcome	Sleep duration<br>β (95% CI)	P-value	FDR
ZME (Memory)	-0.069 (-0.19, 0.05)	0.257	0.533
ZEX (Executive)	-0.043 (-0.13, 0.05)	0.355	0.533
ZVS (Visuospatial)	-0.070 (-0.38, 0.24)	0.657	0.788
ZLA (Language)	-0.008 (-0.13, 0.11)	0.902	0.902
ZAS (Attention-speed)	-0.093 (-0.21, 0.02)	0.111	0.533
ZCO (Composite)	-0.078 (-0.24, 0.08)	0.335	0.533
	Sleep Variability<br>β (95% CI)	P-value	FDR
ZME (Memory)	-0.084 (-0.26, 0.09)	0.347	0.740
ZEX (Executive)	-0.016 (-0.15, 0.12)	0.816	0.816
ZVS (Visuospatial)	-0.149 (-0.60, 0.30)	0.520	0.779
ZLA (Language)	0.028 (-0.15, 0.20)	0.753	0.816
ZAS (Attention-speed)	-0.081(-0.25, 0.09)	0.346	0.740
ZCO (Composite)	-0.107 (-0.34, 0.13)	0.370	0.740

	Efficiency<br>β (95% CI)	P-value	FDR
ZME (Memory)	0.012 (-0.00, 0.03)	0.151	0.903
ZEX (Executive)	0.006 (-0.01, 0.02)	0.358	0.916
ZVS (Visuospatial)	-0.002 (-0.05, 0.04)	0.916	0.916
ZLA (Language)	0.003 (-0.01, 0.02)	0.731	0.916
ZAS (Attention-speed)	0.004 (-0.01, 0.02)	0.597	0.916
ZCO (Composite)	0.002 (-0.02, 0.02)	0.832	0.916
	WASO<br>β (95% CI)	P-value	FDR
ZME (Memory)	-0.007 (-0.01, 0.00)	0.080	0.482
ZEX (Executive)	-0.001 (-0.01, 0.00)	0.612	0.735
ZVS (Visuospatial)	0.011 (-0.01, 0.03)	0.253	0.507
ZLA (Language)	-0.001 (-0.01, 0.01)	0.848	0.848
ZAS (Attention-speed)	-0.005 (-0.01, 0.00)	0.214	0.507
ZCO (Composite)	0.004 (-0.01, 0.01)	0.497	0.735
	Number of Awakenings<br>β (95% CI)	P-value	FDR
ZEX (Executive)	-0.004 (-0.01, 0.00)	0.198	0.395
ZVS (Visuospatial)	-0.003 (-0.02, 0.02)	0.813	0.813
ZLA (Language)	-0.005 (-0.01, 0.00)	0.279	0.419
ZCO (Composite)	-0.002 (-0.01, 0.01)	0.708	0.813
	Onset<br>β (95% CI)	P-value	FDR
ZME (Memory)	-0.001 (-0.00, 0.00)	0.568	0.681

ZEX (Executive)	-0.001 (-0.00, 0.00)	0.385	0.577
ZVS (Visuospatial)	-0.005 (-0.01, 0.01)	0.350	0.577
ZLA (Language)	0.001 (-0.00, 0.00)	0.768	0.768
ZAS (Attention-speed)	-0.002 (-0.01, 0.00)	0.248	0.577
ZCO (Composite)	-0.003 (-0.01, 0.00)	0.222	0.577

Supplementary table S4. Cross-sectional associations between categorical sleep measures and cognitive domain test z-scores in total sample.

Outcome	Normal	Sleep Duration<br>Short<br>β (95% CI)	P-value	FDR	Long<br>β (95% CI)	P-value	FDR
ZME (Memory)	Ref	0.09 (-0.23, 0.41)	0.579	0.897	-0.08 (-0.63, 0.48)	0.790	0.897
ZEX (Executive)	Ref	0.194 (-0.05, 0.44)	0.118	0.753	0.046 (-0.38, 0.47)	0.831	0.897
ZVS (Visuospatial)	Ref	0.219 (-0.61, 1.04)	0.603	0.897	0.372 (-1.04, 1.79)	0.606	0.897
ZLA (Language)	Ref	-0.031 (-0.35, 0.29)	0.852	0.897	-0.149 (-0.70, 0.40)	0.597	0.633
ZAS(Attention-speed)	Ref	0.137 (-0.17, 0.44)	0.380	0.897	-0.409 (-0.93, 0.11)	0.125	0.753
ZCO (Composite)	Ref	0.239 (-0.19, 0.67)	0.274	0.897	0.049 (-0.68, 0.78)	0.897	0.897
	Low	Sleep Variability<br>High*<br>β (95% CI)	P-value	FDR			
ZME (Memory)	Ref	-0.138 (-0.44, 0.17)	0.376	0.638			

ZEX (Executive)	Ref	-0.073 (-0.30, 0.16)	0.535	0.642			
ZVS (Visuospatial)	Ref	-0.326 (-1.11, 0.47)	0.415	0.638			
ZLA (Language)	Ref	0.047 (-0.26, 0.35)	0.762	0.762			
ZAS(Attention-speed)	Ref	-0.119 (-0.41, 0.17)	0.425	0.638			
ZCO (Composite)	Ref	-0.216 (-0.62, 0.19)	0.298	0.638			
	High*	Efficiency<br>Low**<br>β (95% CI)	P-value	FDR			
ZME (Memory)	Ref	-0.220 (-0.55, 0.11)	0.192	0.576			
ZEX (Executive)	Ref	0.001 (-0.25, 0.25)	0 .994	0.994			
ZVS (Visuospatial)	Ref	-0.101 (-0.95, 0.75)	0.817	0.994			
ZAS(Attention-speed)	Ref	0.026 (-0.29, 0.34)	0.871	0.994			
ZCO (Composite)	Ref	-0.169 (-0.61, 0.27)	0.455	0.909			

Supplementary Table S5. Longitudinal associations of continuous sleep measures with cognitive domain z-scores.

Outcome	Sleep duration<br>β (95% CI)	P-value	FDR
ZME (Memory)	-0.109 (-0.27, 0.05)	0.168	0.987

ZEX (Executive)	-0.001 (-0.14, 0.14)	0.987	0.987
ZVS (Visuospatial)	0.061 (-0.53, 0.66)	0.840	0.987
ZLA (Language)	0.058 (-0.07, 0.18)	0.363	0.987
ZAS (Attention-speed)	0.045 (-0.11, 0.20)	0.567	0.987
ZCO (Composite)	0.007 (-0.29, 0.30)	0.960	0.987
	Sleep Variability<br>β (95% CI)	P-value	FDR
ZME (Memory)	-0.101 (-0.30, 0.10)	0.323	0.597
ZEX (Executive)	-0.146 (-0.32, 0.03)	0.104	0.358
ZVS (Visuospatial)	-0.275 (-1.03, 0.48)	0.472	0.768
ZLA (Language)	-0.050 (-0.21, 0.11)	0.542	0.768
ZAS (Attention-speed)	-0.119 (-0.32, 0.08)	0.237	0.512
ZCO (Composite)	-0.223 (-0.60, 0.15)	0.239	0.512
	Efficiency<br>β (95% CI)	P-value	FDR
ZEX (Executive)	0.019 (-0.01, 0.05)	0.153	0.736
ZVS (Visuospatial)	0.047 (-0.07, 0.16)	0.411	0.926
ZAS (Attention-speed)	0.026 (-0.00, 0.06)	0.078	0.621
ZCO (Composite)	0.029 (-0.03, 0.09)	0.310	0.926
	WASO<br>β (95% CI)	P-value	FDR
ZEX (Executive)	-0.002 (-0.01, 0.01)	0.642	0.855
ZVS (Visuospatial)	0.019 (-0.01, 0.05)	0.269	0.707
ZLA (Language)	-0.005 (-0.01, 0.00)	0.189	0.707
ZAS (Attention-speed)	-0.002 (-0.01, 0.01)	0.593	0.838

ZCO (Composite)	0.007 (-0.01, 0.02)	0.424	0.707
	Number of Awakenings β (95% CI)	P-value	FDR
ZEX (Executive)	-0.005 (-0.01, 0.00)	0.213	0.920
ZVS (Visuospatial)	-0.004 (-0.04,0.03)	0.808	0.920
ZAS (Attention-speed)	-0.008 (-0.02, 0.00)	0.073	0.439
ZCO (Composite)	-0.003 (-0.02, 0.01)	0.747	0.920
	Onset β (95% CI)	P-value	FDR
ZME (Memory)	-0.005 (-0.01, 0.00)	0.089	0.335
ZEX (Executive)	-0.004 (-0.01, 0.00)	0.098	0.335
ZVS (Visuospatial)	-0.020 (-0.04, 0.00)	0.065	0.318

Supplementary Table S6. Longitudinal associations of categorical sleep measures with cognitive domain z-scores.

Outcome		Sleep Duration					
	Normal	Short β (95% CI)	P-value	FDR	Long β (95% CI)	P-value	FDR
ZME (Memory)	Ref	-0.022 (-0.39, 0.35)	0.906	0.946	-0.533 (-1.14, 0.08)	0.085	0.409
ZEX (Executive)	Ref	0.062 (-0.27, 0.40)	0.712	0.876	0.132 (-0.41, 0.68)	0.633	0.876
ZVS (Visuospatial)	Ref	0.274 (-1.16 , 1.71)	0.704	0.876	0.904 (-1.45, 3.26)	0.447	0.876
ZAS(Attention-speed)	Ref	-0.145 (-0.52,0.23)	0.441	0.876	0.045 (-0.56, 0.65)	0.833	0.946
ZCO (Composite)	Ref		0.766	0.897		0.680	0.876

		0.108 (-0.61, 0.82)			0.246 (-0.93,1.42)		
	Low	Sleep Variability<br>High<br>β (95% CI)	P-value	FDR			
ZME (Memory)<br>ZEX (Executive)<br>ZVS (Visuospatial)<br>ZLA (Language)<br>ZAS(Attention-speed)<br>ZCO (Composite)	Ref<br>Ref<br>Ref<br>Ref<br>Ref<br>Ref	-0.268 (-0.63, 0.09)<br>-0.213 (-0.53, 0.10)<br>-0.835 (-2.18, 0.51)<br>-0.009 (-0.30, 0.28)<br>-0.240 (-0.60, 0.12)<br>-0.546 (-1.21, 0.12)	0.141<br>0.187<br>0.222<br>0.949<br>0.184<br>0.107	0.561<br>0.561<br>0.561<br>0.949<br>0.561<br>0.561			
	High	Efficiency<br>Low<br>β (95% CI)	P-value	FDR			
ZME (Memory)<br>ZEX (Executive)<br>ZVS (Visuospatial)<br>ZLA (Language)<br>ZAS (Attention-speed)<br>ZCO (Composite)	Ref<br>Ref<br>Ref<br>Ref<br>Ref<br>Ref	-0.172 (-0.56, 0.22)<br>-0.038 (-0.38, 0.31)<br>-0.657 (-2.14, 0.82)<br>-0.284 (-0.59, 0.02)<br>-0.035 (-0.42, 0.35)	0.381<br>0.828<br>0.380<br>0.070<br>0.856<br>0.348	0.894<br>0.894<br>0.894<br>0.894<br>0.894<br>0.894			

		-0.349 (-1.08, 0.39)					

Supplementary Table S7. Sleep × time spline interaction p-values for longitudinal cognitive outcome.

Sleep measure	Cognitive outcome	Spline component	β (95% CI)	P-value	FDR
Number of Awakenings	ZME (Memory)	Spline 2	0.002 (-0.02, 0.02)	0.844	0.951
		Spline 3	0.000 (-0.02, 0.02)	0.988	0.988
Sleep Duration	ZLA(Language)	Spline 1	0.126 (-0.15, 0.40)	0.373	0.831
		Spline 3	-0.330 (-0.76, 0.10)	0.127	0.762
Sleep Variability	ZME (Memory)	Spline 1	0.351 (-0.02, 0.72)	0.061	0.194

Supplementary Table S8. Phase-specific trends between sleep measures and cognitive outcomes derived from linear mixed-effects models.

Sleep measure	Cognitive outcome	Time Phase	β (95% CI)	P-value
Sleep Duration	ZLA(Language)	Early	0.058 (-0.06, 0.18)	0.349
		Middle		0.643

		Late	-0.029 (-0.15, 0.09)	0.892
			0.008 (-0.11, 0.13)	
Sleep Variability	ZME (Memory)	Early	-0.117 (-0.33, 0.09)	0.271
		Middle	-0.131 (-0.34, 0.08)	0.224
